# NEIL3-deficiency increases gut permeability and contributes to a pro-atherogenic metabolic phenotype

**DOI:** 10.1038/s41598-021-98820-0

**Published:** 2021-10-05

**Authors:** Tom Rune Karlsen, Xiang Yi Kong, Sverre Holm, Ana Quiles-Jiménez, Tuva B. Dahl, Kuan Yang, Ellen L. Sagen, Tonje Skarpengland, Jonas D. S. Øgaard, Kristian Holm, Beate Vestad, Maria B. Olsen, Pål Aukrust, Magnar Bjørås, Johannes R. Hov, Bente Halvorsen, Ida Gregersen

**Affiliations:** 1 Research Institute of Internal Medicine, Oslo University Hospital, Rikshospitalet, Oslo, Norway; 2 Institute of Clinical Medicine, Faculty of Medicine, University of Oslo, Oslo, Norway; 3 Department of Research and Development, Division of Emergencies and Critical Care, Oslo University Hospital HF, Rikshospitalet, Oslo, Norway; 4 Section of Clinical Immunology and Infectious Diseases, Oslo University Hospital, Rikshospitalet, Oslo, Norway; 5 Section of Gastroenterology, Department of Transplantation Medicine, Oslo University Hospital, Oslo, Norway; 6 Department of Microbiology, Oslo University Hospital, Rikshospitalet, Oslo, Norway; 7 Department of Clinical and Molecular Medicine, Norwegian University of Science and Technology, Trondheim, Norway; 8 Norwegian PSC Research Center, Department of Transplantation Medicine, Oslo University Hospital, Oslo, Norway

**Keywords:** DNA damage and repair, Metabolomics, Atherosclerosis

## Abstract

Atherosclerosis and its consequences cause considerable morbidity and mortality world-wide. We have previously shown that expression of the DNA glycosylase NEIL3 is regulated in human atherosclerotic plaques, and that NEIL3-deficiency enhances atherogenesis in *Apoe*^*−/−*^ mice. Herein, we identified a time point prior to quantifiable differences in atherosclerosis between *Apoe*^*−/−*^*Neil3*^*−/−*^ mice and *Apoe*^*−/−*^ mice. Mice at this age were selected to explore the metabolic and pathophysiological processes preceding extensive atherogenesis in NEIL3-deficient mice. Untargeted metabolomic analysis of young *Apoe*^*−/−*^*Neil3*^*−/−*^ mice revealed significant metabolic disturbances as compared to mice expressing NEIL3, particularly in metabolites dependent on the gut microbiota. 16S rRNA gene sequencing of fecal bacterial DNA indeed confirmed that the NEIL3-deficient mice had altered gut microbiota, as well as increased circulating levels of the bacterially derived molecule LPS. The mice were challenged with a FITC-conjugated dextran to explore gut permeability, which was significantly increased in the NEIL3-deficient mice. Further, immunohistochemistry showed increased levels of the proliferation marker Ki67 in the colonic epithelium of NEIL3-deficient mice, suggesting increased proliferation of intestinal cells and gut leakage. We suggest that these metabolic alterations serve as drivers of atherosclerosis in NEIL3-deficient mice.

## Introduction

Cardiovascular disease (CVD) is the number one cause of morbidity and mortality worldwide. In 2019, an estimated 18.6 million people died from CVD, including 9.14 million from ischemic heart disease, and 6.55 million from cerebral stroke^1^. The main driver of CVD is atherosclerosis, a chronic disease characterized by bidirectional interaction between lipids and inflammation^2^. The disease involves complex interaction between the immune system and metabolic disturbances^3^, but we do not know all the central pathways that are participating in this interplay. Such knowledge is of major importance in order to identify new targets for therapy. One hypothesis that is gaining momentum is that DNA damage, in particular oxidative DNA damage, may play a role in atherogenesis^4,5^. It therefore stands to reason that deficiency in the DNA repair machinery of cells might aggravate atherosclerosis.

The main cellular apparatus for repair of oxidative DNA damage is the base excision repair (BER) pathway^6^. Endonuclease VIII-like 3 (NEIL3) DNA glycosylase is one of the enzymes that initiate this repair process. In a human case–control study we demonstrated that a single nucleotide polymorphism (SNP) in *NEIL3* is associated with increased risk of myocardial infarction^7^. Further, *NEIL3* expression is increased in human atherosclerotic plaques^8^. In addition, we have described increased atherosclerosis in *Apoe*^*−/−*^*Neil3*^*−/−*^ mice compared to *Apoe*^*−/−*^mice, involving macrophage action (high fat diet model)^8^ and altered function of smooth muscle cells (SMCs, chow diet model)^9^. Despite NEIL3’s role in DNA repair, we did not find increased DNA damage in the NEIL3-deficient mice, independently of diet^8,9^. These results suggest a role for NEIL3 in atherogenesis, possibly through mechanisms other than its role in the BER pathway. In fact, increasing evidence suggests that NEIL3 has cellular functions beyond its canonical DNA repair properties^10–12^.

Recently, we showed that NEIL3-deficiency can be linked to transdifferentiation of SMCs in the arterial wall, as well as increased proliferation of these cells^9^. Herein, we perform a detailed study of the metabolic profile of *Apoe*^*−/−*^*Neil3*^*−/−*^ mice on chow diet to further elucidate the underlying mechanism of increased atherosclerosis observed in these mice.

## Results

### Age-dependent differences in atherosclerosis between ***Apoe***^*−/−*^***Neil3***^*−/−*^mice and ***Apoe***^*−/−*^mice

**Figure 1:**
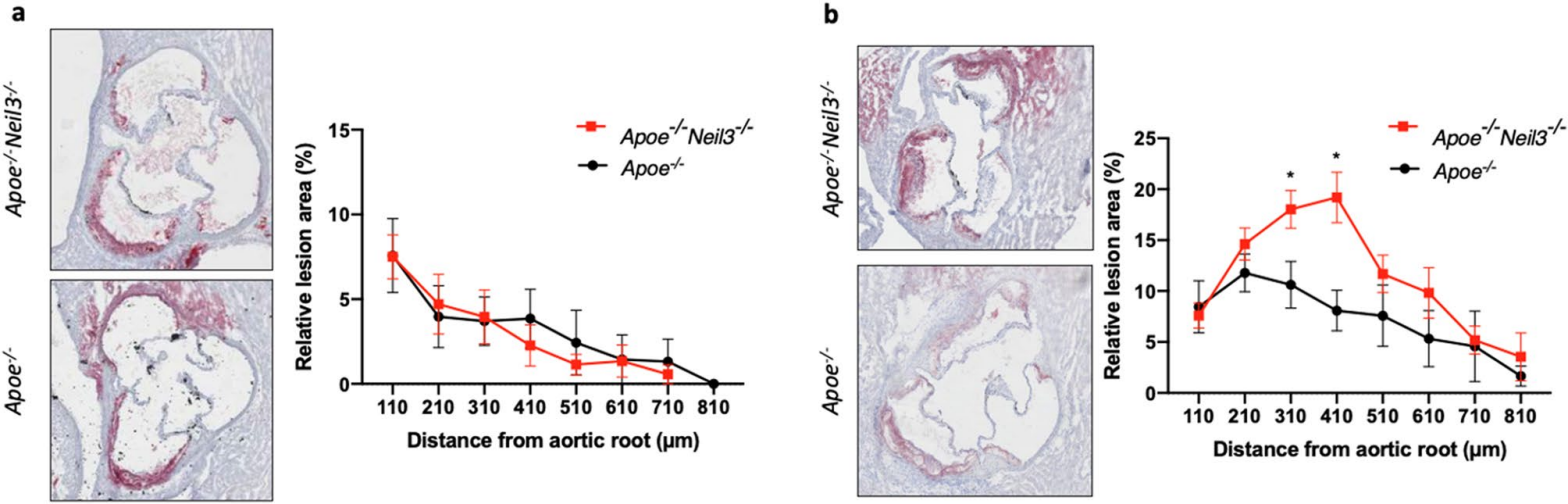
Age-dependent differences in atherosclerosis in *Apoe*^*−/−*^*Neil3*^*−/−*^ mice as compared to *Apoe*^*−/−*^ mice. Representative cryosections at 310 µm distance from the aortic root, stained with Oil Red O from *Apoe*^*−/−*^*Neil3*^*−/−*^ and *Apoe*^*−/−*^ mice at (**a**) 16 and (**b**) 24 weeks of age. The relative lesion area from each cryosection (plaque area/aorta circumference × 100), taken at 100 µm intervals from the aortic root. Data are presented as mean ± SEM, Student’s *t* test, n = 4–6, *p < 0.05.

We have previously shown that NEIL3-deficient mice develop more atherosclerosis than control mice at a mature age^8,9^. To better understand the events preceding development of this phenotype, we investigated atherosclerosis development in a set of younger mice. In this study, we therefore included two groups of mice, one with similar age to our previous studies (24 weeks), and one group with younger mice (16 weeks), both on a standard chow diet. We did not see any differences in atherosclerosis in younger mice when comparing *Apoe*^*−/−*^*Neil3*^*−/−*^ mice to *Apoe*^*−/−*^ mice (Fig. 1a). In older mice, increased atherosclerosis in the *Apoe*^*−/−*^*Neil3*^*−/−*^ genotype compared to the *Apoe*^*−/−*^ genotype was confirmed, suggesting a shift in phenotype between 16 and 24 weeks of age (Fig. 1b). The 16-week-old mice were therefore chosen for further experiments to elucidate the underlying mechanisms of NEIL3-deficiency-driven atherosclerosis.

### NEIL3-deficiency has significant impact on the metabolome of ***Apoe***^*−/−*^ mice

**Figure 2:**
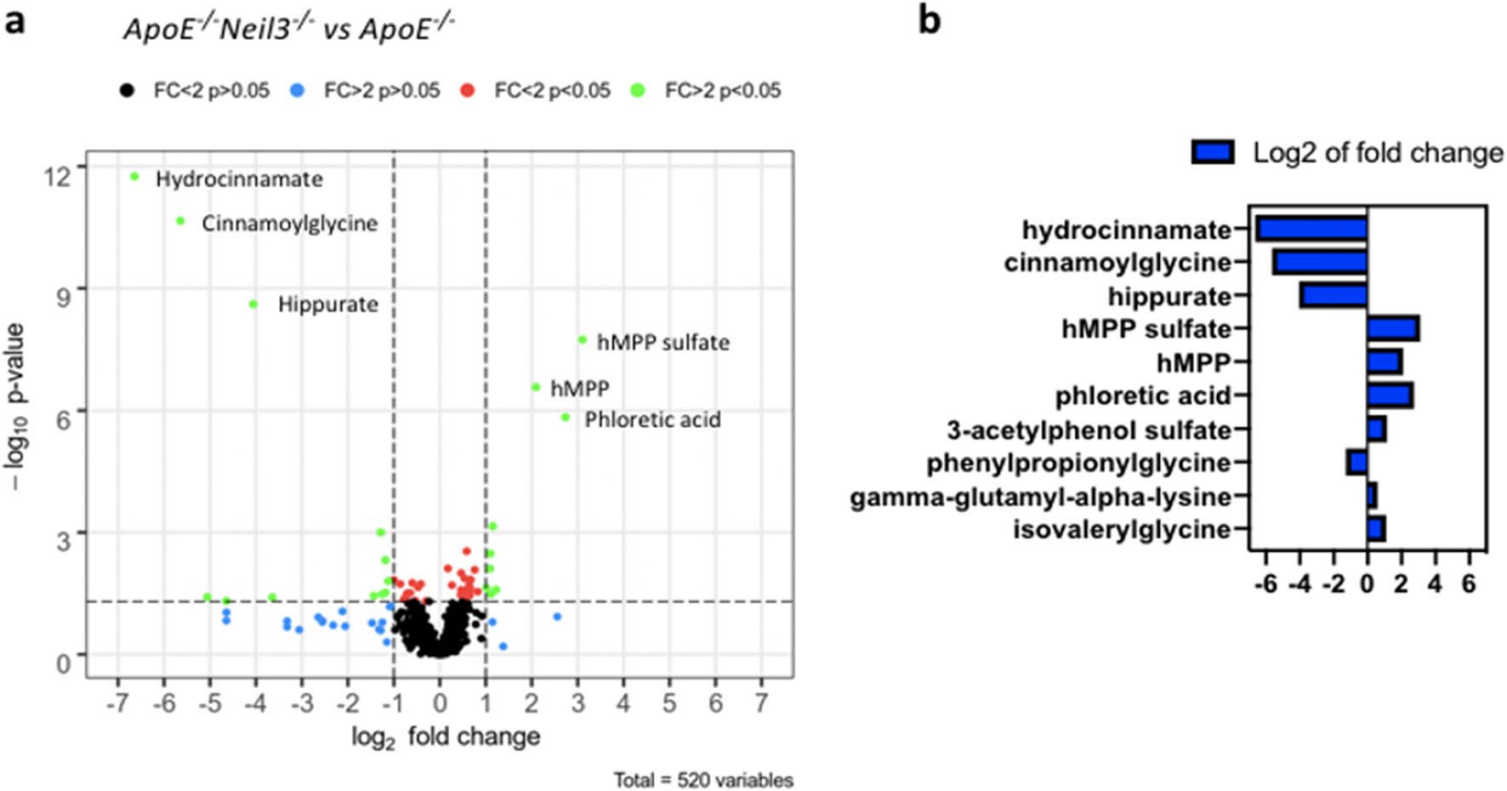
Different levels of plasma metabolites in *Apoe*^*−/−*^*Neil3*^*−/−*^ mice as compared to *Apoe*^*−/−*^ mice. (**a**) Volcano plot displaying plasma metabolites differentially expressed between *Apoe*^*−/−*^*Neil3*^*−/−*^ and *Apoe*^*−/−*^ mice. The log2 fold change of each individual metabolite is represented on the x-axis. The y-axis corresponds to the − log10 of the *p* value. The dashed lines indicate the cutoff for *p* value (0.05) and fold change (twofold), and significant metabolites are presented as green dots. (**b**) Top 10 significant metabolites in *Apoe*^*−/−*^*Neil3*^*−/−*^ as compared to *Apoe*^*−/−*^ mice, ordered by *p* value. hMPP: 3-(3-hydroxyphenyl) propionate. FC: fold change. Two-way ANOVA, n = 5.

Metabolic alterations are important drivers of atherogenesis. We have previously demonstrated that NEIL3-deficient mice display lipid disturbances upon high fat feeding^8^. However, a thorough metabolic characterization of NEIL3-deficient mice on a chow diet has not been performed. To explore the metabolic events preceding the development of extensive atherosclerosis, we analyzed a comprehensive metabolomic panel of plasma samples from *Apoe*^*−/−*^*Neil3*^*−/−*^ mice and *Apoe*^*−/−*^ mice at 16 weeks of age. Among 520 analyzed metabolites, 22 of them displayed a minimum of twofold significantly different expression between the genotypes (Fig. 2a). Within the most regulated metabolites were several that are involved in the catabolic pathways from aromatic amino acids and polyphenols to benzoate, including 3-phenylpropionic acid, cinnamoylglycine and 3-(4-hydroxyphenyl) propionate, as well as the glycine conjugate of benzoate, hippurate (Fig. 2b). The catabolic pathways of aromatic amino acids are heavily dependent on gut microbiota, since mammals lack the necessary enzymes for breakdown of these substrates^13–15^. Also, butyrate, a short-chain fatty acid with significant impact on host metabolism^16^, was decreased in the *Apoe*^*−/−*^*Neil3*^*−/−*^ mice as compared to controls (fold change 0.08, *p* value 0.04). Butyrate is in part produced by gut bacteria^17^. These results could therefore suggest differences in gut microbiota composition between the genotypes.

### ***Apoe***^*−/−*^***Neil3***^*−/−*^ mice display significant gut microbiota alterations

**Figure 3:**
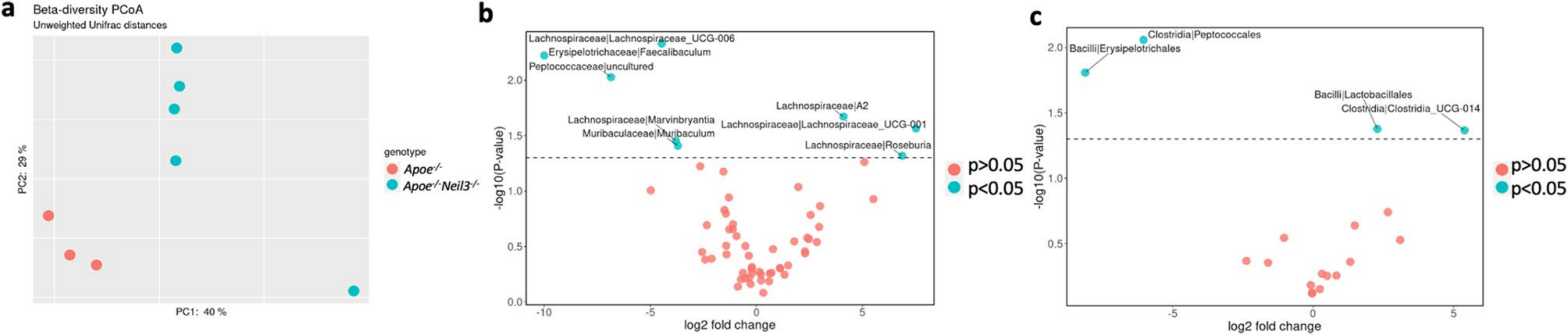
Significant differences in gut microbiota between *Apoe*^*−/−*^*Neil3*^*−/−*^ and *Apoe*^*−/−*^ mice. (**a**) Bacterial beta diversity represented by Principal Coordinate Analysis (PCoA) plot, showing the distance of colonic microbial communities in *Apoe*^*−/−*^*Neil3*^*−/−*^ as compared to *Apoe*^*−/−*^ mice (permutational multivariate analysis of variance, *p* = 0.02). Red dots, *Apoe*^*−/−*^ mice; blue dots, *Apoe*^*−/−*^*Neil3*^*−/−*^ mice. (**b**) Significant differences (blue dots, *p* < 0.05) in the intestinal microbiota of *Apoe*^*−/−*^*Neil3*^*−/−*^ mice as compared to *Apoe*^*−/−*^ mice (genus level, Aldex2). (**c**) Significant differences (blue dots, *p* < 0.05) in the intestinal microbiota of *Apoe*^*−/−*^*Neil3*^*−/−*^ mice as compared to *Apoe*^*−/−*^ mice (order level, Aldex2). n = 3–5.

Based on our observed differences in plasma metabolites, we next investigated the gut microbiota composition in *Apoe*^*−/−*^*Neil3*^*−/−*^ mice and *Apoe*^*−/−*^ mice, which was found to differ significantly between the genotypes (Fig. 3a). Of particular interest, at genus level, *Faecalibaculum* was decreased in NEIL3-deficient mice, while *Roseburia* was increased (Fig. 3b). Alterations were also found at the order level (Fig. 3c). Of note, the order Lactobacillales was more abundant in the *Apoe*^*−/−*^*Neil3*^*−/−*^ mice as compared to *Apoe*^*−/−*^ mice. No Proteobacteria were present in either of the genotypes.

### Increased proliferation of colonic epithelial cells in NEIL3-deficient mice

**Figure 4:**
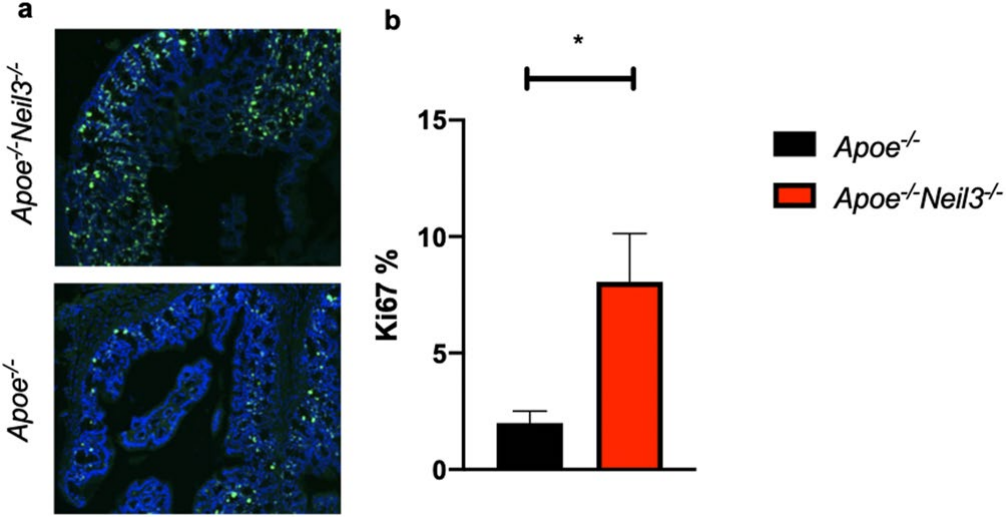
Increased expression of Ki67 in NEIL3-deficient colonic epithelial cells. (**a**) Representative immunohistochemistry images of large bowel sections from *Apoe*^*−/−*^*Neil3*^*−/−*^ and *Apoe*^*−/−*^ mice stained with nuclei marker DAPI (blue) and proliferation marker anti-Ki67 (green). (**b**) Relative abundance of Ki67-positive cells. Data are presented as mean ± SEM, Student’s *t* test, *p < 0.05, n = 5.

The gut microbiota composition influences the rate of colonic epithelial cell proliferation^18,19^. As NEIL3 has a known effect on cellular proliferation^9,20^, this led us to investigate the level of proliferation in the epithelial cells of the large bowel of *Apoe*^*−/−*^*Neil3*^*−/−*^ and *Apoe*^*−/−*^ mice. Histological sections of the colon showed significantly higher staining for the proliferation marker Ki67 in the *Apoe*^*−/−*^*Neil3*^*−/−*^ mice, suggesting increased cellular proliferation in the bowel epithelial cells of these mice (Fig. 4).

### Increased intestinal permeability in NEIL3-deficient mice

**Figure 5:**
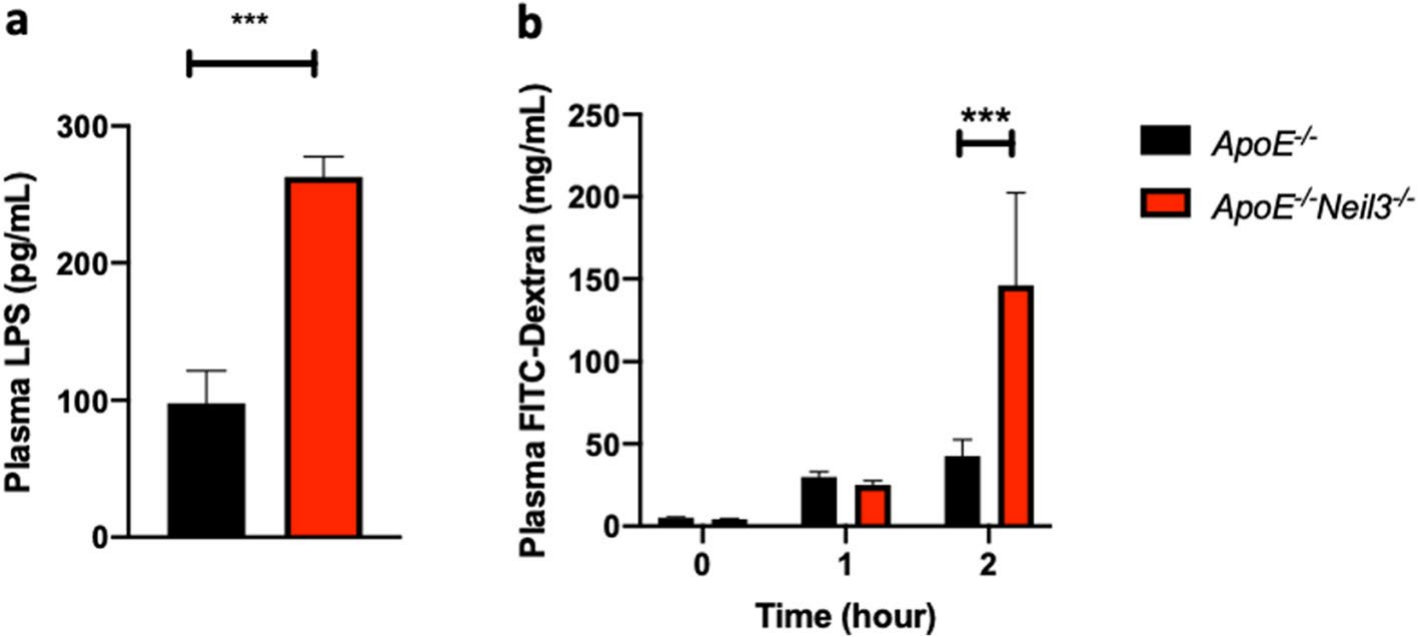
Increased gut permeability in NEIL3-deficient mice. (**a**) Plasma levels of lipopolysaccharide (LPS). (**b**) Plasma levels of FITC-dextran measured in *Apoe*^*−/−*^*Neil3*^*−/−*^ and *Apoe*^*−/−*^ mice 1 and 2 h after oral administration. Data are presented as mean ± SEM, Student’s *t* test, ***p < 0.001, n = 5–8.

Increased intestinal epithelial cell proliferation has been linked to increased gut permeability in mice^21,22^. It has also been shown that the gut microbiota composition affects the permeability of the intestinal wall^23,24^, and studies have shown that increased intestinal permeability is associated with atherosclerosis and CVD^25^. We therefore measured lipopolysaccharide (LPS) in the plasma of the mice as an established marker of impaired gut integrity^26–29^. LPS was significantly increased in the *Apoe*^*−/−*^*Neil3*^*−/−*^ mice as compared to the *Apoe*^*−/−*^ mice, suggesting enhanced gut leakage in NEIL3-deficient *Apoe*^*−/−*^ mice (Fig. 5a).

To further examine the intestinal permeability in the two genotypes, mice were fed fluorescein isothiocyanate (FITC)-labeled dextran after 4 h of fasting. We found a significant increase in the uptake of this carbohydrate after 2 h in *Apoe*^*−/−*^*Neil3*^*−/−*^ mice as compared to *Apoe*^*−/−*^ mice, confirming increased gut permeability in *Apoe*^*−/−*^*Neil3*^*−/−*^ mice (Fig. 5b).

**Figure 6:**
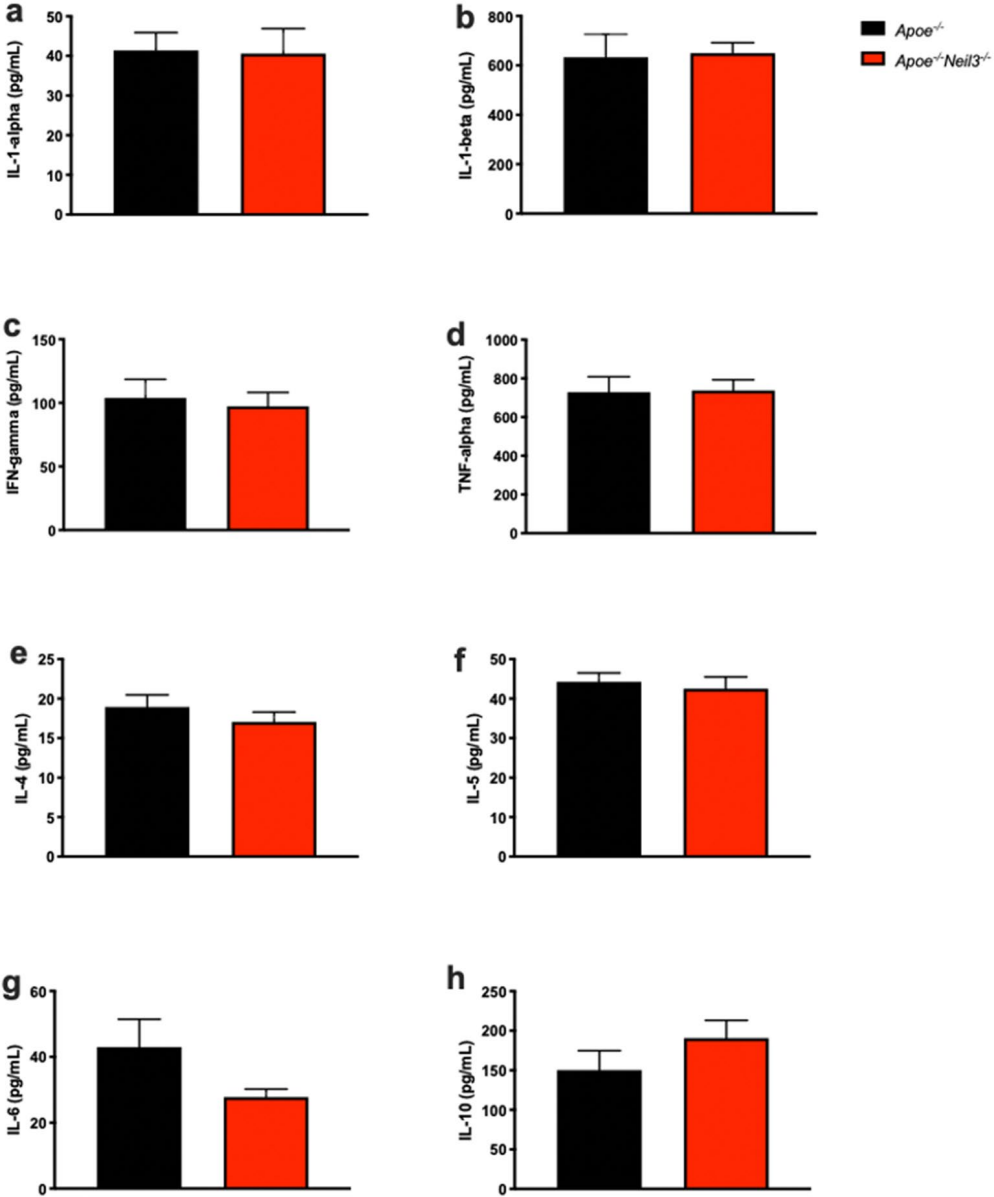
Non-significant difference of plasma cytokine levels between *Apoe*^*−/−*^*Neil3*^*−/−*^ mice and *Apoe*^*−/−*^ mice. (**a**) IL 1-alpha. (**b**) IL 1-beta. (**c**) IFN-gamma. (**d**) TNF-alpha. (**e**) IL-4. (**f**) IL-5. (**g**) IL-6. (**h**) IL-10. Data are presented as mean ± SEM, *t* test, n = 5–6. IL: interleukin, IFN: interferon, TNF: tumor necrosis factor.

Intestinal leakage of LPS is known to induce systemic inflammation^30^, so we expected to find increased levels of pro-inflammatory cytokines in the *Apoe*^*−/−*^*Neil3*^*−/−*^ mice. Surprisingly, this was not the case, as these cytokines showed similar plasma levels between the genotypes (Fig. 6).

## Discussion

In this study we explore the metabolic events preceding development of extensive atherosclerosis in *Apoe*^*−/−*^*Neil3*^*−/−*^ and *Apoe*^*−/−*^ mice to further map the role of NEIL3 in atherogenesis. At 16 weeks of age, there were no differences in atherosclerosis between the genotypes, and these young mice were studied further. At this age, *Apoe*^*−/−*^*Neil3*^*−/−*^ mice exhibited substantial metabolic alterations compared to *Apoe*^*−/−*^ mice, accompanied by altered gut microbiota composition, increased gut permeability and increased plasma LPS levels in *Apoe*^*−/−*^*Neil3*^*−/−*^ mice.

The metabolic panel reveals several striking differences in the metabolome between the genotypes. Notably hippurate, the final common product of aromatic amino acid breakdown, was present in 20-fold lower plasma concentrations in the *Apoe*^*−/−*^*Neil3*^*−/−*^ mice as compared to the *Apoe*^*−/−*^ mice. Hippurate is shown to have a negative correlation with obesity and features of the metabolic syndrome in humans^31,32^. Metabolic syndrome is a risk factor for atherosclerotic disease, but the role of hippurate in atherogenesis is not known and the consequence of reduced levels of hippurate herein needs further investigation.

The gut microbiota was found to differ significantly between the genotypes. At genus level, *Faecalibaculum* was decreased, and at order level, Lactobacillales was increased, when comparing *Apoe*^*−/−*^*Neil3*^*−/−*^ mice to *Apoe*^*−/−*^ mice. *Faecalibaculum* has been negatively correlated with metabolic markers of atherosclerosis^33^, and Lactobacillales has been found to correlate positively with coronary artery disease (CAD) in humans^34^. These associations have not been demonstrated to have a causal relationship. Our results, however, suggest that the gut microbiota changes precede, rather than follow, extensive development of atherosclerosis, making a cause-and-effect relationship possible.

At the junction between the gut bacteria and the host cells is the colonic mucus layer, secreted by the host epithelial cells. The mucus layer affects the composition of the microbiota, at least in part by functioning as an energy source for the bacteria^35,36^. This relationship also works in reverse, in that bacterial metabolites can be an energy source for the colonic epithelial cells^17^. Our data showed increased expression of Ki67, a marker of cell proliferation, in the colon of *Apoe*^*−/−*^*Neil3*^*−/−*^ mice, suggesting different energy demand between the genotypes. Butyrate, a bacterial metabolite, was found to be decreased in the plasma of *Apoe*^*−/−*^*Neil3*^*−/−*^ mice. Butyrate deficiency has been linked to increased gut permeability and increased atherosclerosis^37^, the latter effect probably mediated through decreased cholesterol efflux in macrophages^38^. This is in agreement with our previous results, where we reported decreased cholesterol efflux in macrophages from *Apoe*^*−/−*^*Neil3*^*−/−*^ mice^8^. Further, NEIL3 is strongly involved in cell proliferation, and we hypothesize that increased cellular turnover in the colonic epithelial cells of NEIL3-deficient mice leads to increased cellular shedding and/or impaired cellular maturation, changing the composition of the colonic mucus layer. In turn, this contributes to shaping of the gut microbiota composition.

Increased plasma LPS in the NEIL3-deficient mice further suggests a link between our observations in the gut of the mice and development of atherosclerosis, as the NEIL3-deficient mice develop extensive atherosclerosis with age. While an association between elevated LPS levels and risk for atherosclerosis is established^39^, the precise mechanism is unknown. We investigated the possibility of increased systemic inflammation in the NEIL3-deficient mice, but surprisingly, we found no increase in pro-inflammatory cytokines in the *Apoe*^*−/−*^*Neil3*^*−/−*^ mice. Studies have shown that different types of LPS have different immunogenicity, depending on their acetylation status^40,41^, and the lack of the typical pro-inflammatory Proteobacteria in the gut microbiota may suggest that the LPS in our mouse models are mainly anti-inflammatory penta-acylated molecules. Proposed non-inflammatory mechanisms linking LPS and atherosclerosis include low-density lipoprotein (LDL) oxidation^42^, increased lipid uptake in adventitial fibroblasts^43^, and direct endothelial cell toxicity^44–46^. LPS is also implicated as an inducer of SMC proliferation, possibly through the Akt pathway^47^. This is in agreement with our previous results, where we described increased proliferation of SMCs through this pathway in *Apoe*^*−/−*^*Neil3*^*−/−*^ mice^9^. Further, the fact that we found no alterations in levels of inflammatory cytokines, and thus no systemic inflammation, also fits with our previous data in this mouse model, showing only minor and non-consistent changes in inflammatory cytokines. Our data may suggest potential pro-atherogenic effects of LPS beyond its role in inflammation. Further, intestinal leakage was confirmed with an in vivo experiment, establishing that our mouse model exhibits a pathological gut permeability. Taken together with our finding of increased levels of the cell proliferation marker Ki67, this agrees with previous reports of leaky gut accompanying increased intestinal epithelial cell proliferation^21,22^.

**Figure 7:**
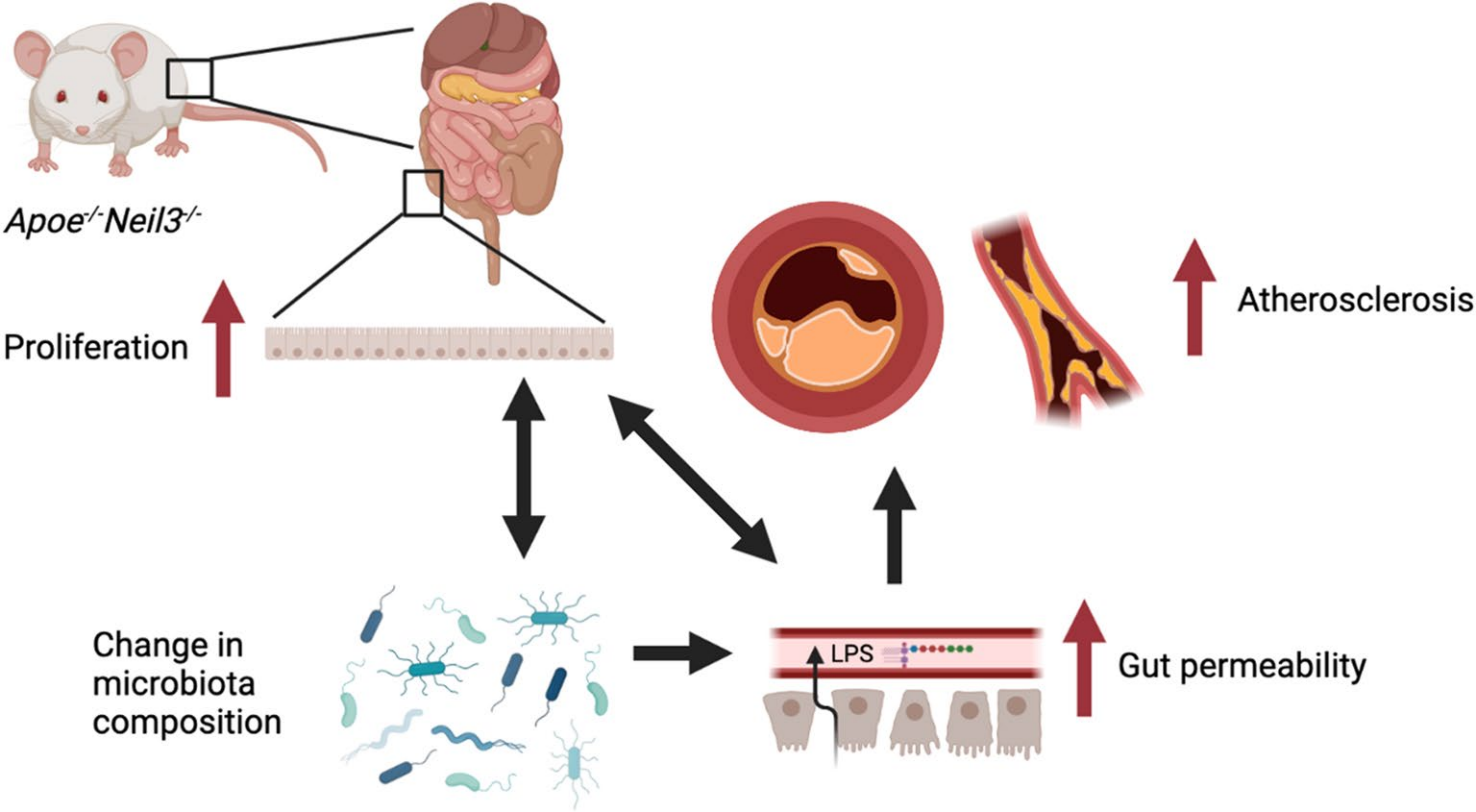
Graphical abstract. Our results show increased proliferation of colon epithelial cells, altered gut microbiota composition and increased gut permeability in young *Apoe*^*−/−*^*Neil3*^*−/−*^ mice as compared to *Apoe*^*−/−*^ mice. We suggest these findings as drivers of accelerated atherosclerosis development which these mice develop with age. Figure created with BioRender.com.

In summary, we report that young *Apoe*^*−/−*^*Neil3*^*−/−*^ mice have a significantly altered metabolome as well as an altered gut microbiota composition as compared to *Apoe*^*−/−*^ mice. This is coupled with increased colonic epithelial cell proliferation, increased intestinal permeability, and increased plasma LPS levels in NEIL3-deficient mice. We propose that these effects on metabolism, gut microbiota and gut leakage could contribute to the enhanced atherosclerosis observed in old *Apoe*^*−/−*^*Neil3*^*−/−*^ mice, as summarized in Fig. 7. We hypothesize that a similar pathogenic interplay may occur in humans, as alterations in gut microbiota have been implicated in atherogenesis also in the human setting^48^. We have previously demonstrated an association between a polymorphism in NEIL3 and increased risk of myocardial infarction^7^, as well as increased expression of NEIL3 in human atherosclerotic plaques^8^, supporting a role for NEIL3 in human atherogenesis. The connection between NEIL3 and gut microbiota and its association with atherosclerosis in humans, however, need further investigation.

This study adds important knowledge to our previous reports on the role of NEIL3 in atherogenesis^8,9^, revealing a multi-faceted role for NEIL3 during the development of atherosclerosis in mice, including effects on lipid metabolism, cell proliferation and cell transdifferentiation, as well as modulation of the gut microbiota and gut health. In future studies we will aim to reveal how these effects are integrated, ultimately leading to increased risk of atherosclerosis.

This study has some limitations. The number of individuals was relatively low in some experiments. Moreover, the lack of co-housing of genotypes, which is preferable for microbiota analysis, may have influenced our results. Also, the inflammatory markers were measured only in plasma and analyses of circulating immune cells could have given additional information. Finally, the study has not pinpointed the molecular mechanism leading to the described phenotype and further mechanistic studies are warranted.

## Materials and methods

### Animal models

NEIL3-deficient (*Neil3*^*−/−*^) mice were generated by germline deletion of exons 3–5 as described by Sejersted et al.^49^, followed by backcrossing into C57BL/6 mice for 10 generations. Then, *Apoe*^*−/−*^*Neil3*^*−/−*^ mice were generated by crossing *Neil3*^*−/−*^ mice with *Apoe*^*−/−*^ mice (C57BL/6 background), obtained from Taconic (Denmark). Mice do not spontaneously develop atherosclerosis unless challenged with a Western diet for a very long time, and murine models with genetically modified lipid metabolism has been the gold standard of model systems to study atherogenesis. Herein, Apoe^*−/−*^ background was chosen based on its ability to develop atherosclerosis without diet intervention^50^. Age- and gender-matched mice were born at the Centre for Comparative Medicine, Oslo University Hospital Rikshospitalet, Oslo (Norway). The mice were fed a standard chow diet, with standard concentrations of lipids and cholesterol (RM3-P, product code 801700) from Special Diets Services (www.sdsdiets.com) ad libitum. Mice used in the same experiments were co-housed, born at the same time, and placed in cages in the same room in a randomized manner. This study has been approved by the Norwegian National Animal Research Authority with project license numbers FOTS 22322, FOTS 8648 and FOTS 23929. All animal experiments were performed in accordance with the European Directive 2010/63/EU and in compliance with relevant ARRIVE guidelines.

### Mouse tissue collection

Organ and tissue harvest took place under non-fasting conditions at 16 and 24 weeks of age. The mice were anaesthetized with 4% isoflurane in an induction chamber, before application of a mask with 2% isoflurane. Blood was collected by trans-thoracic cardioscentesis, using a 1 mL syringe with coating of 0.5 mol/mL EDTA (Fluka, Sigma-Aldrich), and the mice were euthanized by exsanguination. EDTA blood was immediately placed on ice and centrifuged within 30 min at 2000*g* (4 °C) for 20 min to obtain plasma. The plasma was kept at − 80 °C until analysis. Cranial halves of the hearts were placed in optimal cutting temperature (OCT) compound (Tissue-Tek, Sakura Finetek, Torrance, the USA) and frozen at − 80 °C until analysis. The large bowel was dissected and flushed with phosphate-buffered saline (PBS) to remove feces. The feces were snap frozen in liquid nitrogen and subsequently kept at − 80 °C until analysis. The cleaned large bowel was kept on 4% formalin at 4 °C until further processing.

### Histological analysis of the aortic root and colon

Frozen hearts in OCT compound (Tissue-Tek, Sakura Finetek, Torrance, the USA) were sectioned from the caudal to the cranial direction at 10 µm intervals on a cryostat. The sections were collected, starting at 90 µm distance after the appearance of the aortic cusps, and stopping at 880 µm. Great caution was taken to prevent oblique sections. Sections were air-dried and fixated with 4% paraformaldehyde (PFA). PFA-fixed sections collected at 100 µm intervals were stained with Oil-red-O staining (ORO, Sigma-Aldrich) and hematoxylin (Vector Laboratories). Stained sections were scanned (Axio Scan.Z1, Zeiss, Oberkochen, Germany) and imported to “z9”, an in-house slide storage system tailored for evaluation and quantitative examination of histological sections. Relative plaque area (area of plaque/area of aortic vessel lumen) was determined digitally in 8 consecutive sections. During the determination of the plaque sizes, the samples were blinded to the investigator. Then mean relative plaque area was calculated for each corresponding section for each of the groups of mice.

Formalin-fixated paraffin-embedded colon specimens were sectioned (5 µm), deparaffinized and incubated with rat monoclonal anti-Ki67 antibody (eBioscience, 14-5698-82, dilution 1:2000). Anti-rat antibody (Vector laboratories, MP-7444) conjugated to horseradish peroxidase (HRP) was used as secondary antibody, and Ki67-positive cells were visualized using the HRP substrate Tyramide iFluora488 (AAT Bioquest). Samples were mounted in Slow Fade Gold antifade reagent (Invitrogen), containing diamidino-2-phenylindole (DAPI, Invitrogen, Lot 2210345) for staining of nuclei.

Stained sections were scanned (Axio Scan.Z1, Zeiss, Oberkochen, Germany) and imported to z9. Ki67-positive areas were quantified as percentage coverage of total colon tissue area present on the slide.

### Measurement of plasma metabolites

Metabolomic analysis was performed on plasma samples (Metabolon Inc., Seattle, the USA) using untargeted, ultrahigh-performance liquid chromatography-tandem mass spectrometry (UPLC-MS/MS) as previously described^51^. Briefly, methanol was added to remove protein and recover small metabolites, followed by centrifugation. The resulting extract was divided into five fractions, whereupon four of these fractions were dried and reconstituted in different solvents for analysis in UPLC-MS/MS positive and negative ion modes. Individual compounds were identified by comparison to a metabolite library maintained by Metabolon, based on retention index, mass to charge ratio and chromatographic data.

### Extraction of fecal bacterial DNA and library preparation

Fecal bacterial DNA was extracted as previously described^52^. Briefly, 1–2 fecal pellets were resuspended in lysis buffer containing 20 mg/mL lysozyme (Sigma) and incubated for 30 min at 37 °C. Further lysis was performed by adding 10% SDS and proteinase K, and samples were homogenized using zirconium beads (0.1 mm, BioSpec) in a bead beater, followed by phenol/chloroform extraction method combined with DNA clean-up with the DNeasy Blood and Tissue extraction kit (Qiagen). Initial DNA concentration measurement was performed using DeNovix DS-11 spectrophotometer. DNA libraries were prepared as previously described^53^. Briefly, PCR amplicons targeting the hypervariable regions V3 and V4 of the 16S rRNA gene were generated using dual-indexed universal primers (319F and 806R) and Phusion High-Fidelity PCR Master mix m/HF buffer (Thermo Fisher Scientific, USA). Cleaning, normalization and pooling of PCR products were performed using the SequalPrep Normalization Plate Kit (Thermo Fisher Scientific, USA). Quality control and quantification of pooled libraries were performed using Agilent Bioanalyzer (Agilent Technologies, USA) and Kapa Library Quantification Kit (Kapa Biosystems, London, UK). Sequencing was performed at the Norwegian Sequencing Centre (Oslo, Norway), applying the Illumina MiSeq platform and v3 kit (Illumina, San Diego, CA, USA), set at 300 base pair paired-end reads.

### Sequence processing and bioinformatics

Paired-end reads containing Illumina Universal Adapters or PhiX were discarded using bbduk version 38.90 (BBTools, https://jgi.doe.gov/data-and-tools/bbtools/) (parameters adaptor filter: k = 23 hdist = 1 tbo cf = TRUE ftm = 5. parameters phix filter: k = 31 hdist = 1) and the remaining reads were demultiplexed using cutadapt version 3.3^54^ (parameters:-e 0.1—no-indels—overlap 12—discard-untrimmed—action none). Trimming of indexes, heterogeneity spacers and primers was also done with cutadapt (parameters: -e 0.1—overlap 20—discard-untrimmed—m 250) and the paired-end reads were subsequently quality trimmed and merged using bbmerge version 38.90^55^ (parameters: qtrim = r trimq = 15 maxlength = 440 mininsert = 390). The merged contigs were trimmed to 400 bp and denoised to ASVs (Amplicon Sequence Variants) with deblur^56^ in Qiime2 version 2021.2^57^. Taxonomic classification of ASVs was done based on RESCRIPt^58^ in Qiime2 using a naïve Bayes classifier^59^ trained on the V3–V4 region of a preclustered version (99% sequence similarity) of the Silva database version 138^60^.

Filtering of contaminants was done with the R package microDecon^61^ based on a negative extraction control sample, and ASVs from mitochondria, chloroplast or with lacking taxonomic annotation on order level were manually removed. A de-novo phylogenetic tree was built in Qiime2 based on the remaining ASVs. To reduce the effect of uneven sequencing depths, samples were rarefied (subsampled without replacement) to an even level of 1424 counts per sample, and all further analyses (except differential abundance analysis with Aldex2^62^) were performed on this rarefied dataset. Beta diversity metrics (unweighted UniFrac) were calculated in Qiime2.

### Measurement of plasma LPS

Plasma LPS levels were measured using a commercially available Limulus Amebocyte Lysate (LAL) chromogenic endpoint assay (Lonza, QCL-1000). 10 µL of plasma was diluted fivefold with endotoxin-free water, then heat-inactivated (68 °C) for 12 min to inactivate potential interfering plasma proteins. Results were determined using a standard curve, and units were converted to pg/mL.

### Assessment of in vivo intestinal permeability

The mice were fasted for 4 h before the intestinal permeability experiment. Pre-test blood samples were taken from the calf vein of the animals. The mice were then fed 600 mg/kg bodyweight FITC-dextran (Sigma-Aldrich) by gavage. Blood samples were collected into EDTA-coated tubes (MICROVETTE CB300, Sarstedt) 1 and 2 h after FITC-dextran administration from the calf vein. The blood was kept on ice and centrifuged within 30 min at 2000*g* (4 °C) for 20 min to obtain plasma. The plasma was diluted 1:15 in PBS, and 100 µL of the dilution was transferred to a black 96-well plate. The fluorescence of each sample was determined at 530 nm with excitation at 485 nm. The concentration of FITC-dextran was calculated using a standard curve.

### Statistical analyses

The metabolomics platform was analyzed by Metabolon Inc using the two-way ANOVA test. The microbiota beta-diversity comparison was done using a permutational multivariate analysis of variance (Permanova) test. The differential abundance of microbiota taxa was analyzed using Aldex2 (ANOVA-Like Differential Expression tool for high throughput sequencing data). All other statistical analyses were done by Student´s t-test, using GraphPad Prism version 9. A *p* value of < 0.05 was considered significant. Statistical correction for multiple testing was not applied due to the small sample size and the exploratory focus of the study. Statistical outliers were identified using Grubb’s test with *α* = 0.05.
